# Are basic and lipophilic chain groups highly required in leishmanicidal quinolines to favor the phagolysosome accumulation?

**DOI:** 10.3389/fchem.2025.1655979

**Published:** 2025-08-21

**Authors:** Angel H. Romero

**Affiliations:** Grupo de Química Orgánica Medicinal, Instituto de Quimica-Biologica, Facultad de Ciencias, Universidad de la República, Montevideo, Uruguay

**Keywords:** phagolysosome, *Leishmania*, log P, ionization constant (pKa), quinolines

## Introduction

A phagolysosome is a cytoplasmic body formed through the fusion of a phagosome with a lysosome during the phagocytosis process (Alexander and Vickerman, 1975). The phagolysosome is characterized by an internal acidic environment (*pH* 4.5–5.0) and an internal temperature of 37°C. This internal acidic condition plays an important role in the intracellular destruction of pathogens via enzymatic hydrolytic degradation (Nguyen and Yates, 2021). This body is crucial for the survival of *Leishmania* parasites within the host cell (Zilberstein, 2021).

*Leishmania* is an intracellular parasite that cycles between the midgut of female sandfly vectors and phagolysosomes of mammalian hosts. The infection initiates with the transformation of the parasite found in the midgut of a sandfly into the flagellated promastigote form. Then the parasites are injected into the human skin during a sandfly blood meal and are rapidly phagocytosed by macrophages, which fuse with lysosomes to form phagolysosomes (Zilberstein, 2021). It is documented that the presence of a chemical component such as lipophosphoglycan (LPG) could be essential in the recognition of promastigote parasite by macrophage cells (Desjardins and Descoteaux, 1997; Moradin and Descoteaux, 2012). Once within the phagolysosome, promastigotes are differentiated to a smaller aflagellated intracellular amastigote form, which is favored by the extremely harsh environment inside the phagolysosome (Berman et al., 1979; Chang and Dwyer, 1976). The parasites at this stage survive and elude the host defense mechanism within the phagolysosome (Chang and Dwyer, 1976; Moradin and Descoteaux, 2012) and then proliferate by binary cell division and invade other macrophages or phagocytic (i.e. dendritic cells) or non-professional phagocytic (i.e. fibroblasts) cells. To elude the host immune defense, *Leishmania* parasites developed a mechanism directed to promote a shift in the macrophage polarization, from a defensive macrophage M1 to an attenuated macrophage M2 (Carneiro et al., 2021; Naderer and McConville, 2008; Tomiotto-Pellissier, et al., 2018), which allows their survival and proliferation inside phagolysosomes. Thus, the phagolysosome emerges as an attractive target for the development of leishmanicidal agents, and it is essential to design chemical structures that will be able to accumulate into the phagolysosome taking advantage of their internal acidic characteristic and highly lipophilic membrane. With this prelude in hand, the present article seeks to show the role of some physicochemical properties [e.g., ionization constant (*pK*_*a*_) and lipophilicity (log *P*)] to favor the accumulation of quinoline systems into the lysosome and to subsequently correlate these parameters with the *in vitro* leishmanicidal response against intracellular amastigotes.

## Importance of basicity and lipophilicity in lysosome accumulation

From a physicochemical point of view, it is possible to predict the ability of a quinoline or any type of compound to accumulate into phagolysosomes and/or analogue organelles (e.g., lysosomes). Trapp et al. (2008) demonstrated, in general terms, the important role of basicity in the accumulation of molecules within the lysosome. They predicted the accumulation of organic compounds within the cell by studying the diffusion from the external solution to the cell organelle (e.g., cytosol, lysosome, or mitochondria) using the Fick–Nernst–Planck equation. In the present analysis, most of the studied compounds were based on quinolines including amodiaquine, chloroquine, quinine, mefloquine, primaquine, quinidine, and quinacrine. The rest of the tested compounds included cycloguanil, artemisinin, halofantrine, and pyrimethamine. The study shows that a high and selective accumulation in lysosomes was found for weak mono- and bivalent bases having intermediate to high values of log *K*_*OW*_. The authors proposed that the selective accumulation into lysosomes over other organelles (e.g., cytosol or mitochondria) can be mediated through an “ion trapping” mechanism, in which the protonation of the basic moiety captures the compounds, forming a more hydrophilic species whose outer diffusion is minimized. Physicochemical properties such as the ionization constant (*pK*_*a*_) and the equilibrium constant (log *K*_*OW*_) are key to understanding the accumulation via “ion trapping.” For monovalent weak bases, the optimal parameter for good lysosome accumulation consists of *pK*_*a*_ and log *K*_*OW*_ values ranging between 6 and 10 and between 0 and 3, respectively. An optimal accumulation was corresponded for bases with a *pK*_*a*_ of 8. For bivalent bases, the optimal *pK*_*a2*_ (aliphatic amine) value ranged between 8 and 10 and the *pK*_*a1*_ value ranged between 4 and 8, whereas the optimal log *K*_*OW*_ value ranged between 3 and 6. Neutral compounds (e.g., artemisinin) showed a negligible accumulation into lysosomes.

**SCHEME 1:**
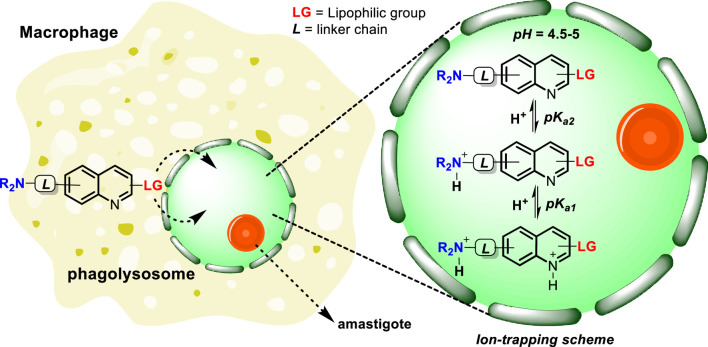
Tentative mechanism of accumulation of quinolines inside the phagolysosome via lipophilic penetration and the subsequent ion trapping mechanism through the protonation of basic moieties in an acidic environment.

Regarding the lipophilicity parameter, Marceau et al. (2012) identified the correlation between the log *P* value and the optimal accumulation into lysosomes. Based on a series of cationic triethylamine derivatives including triethanolamine, procainamide, triethylamine, lidocaine, imatinib, chloroquine, astemizole, quinacrine, dronedarone, and amiodarone, which displayed *pK*_*a*_ values ranging between 8 and 10, compounds with a log *P* value ranging between 1 and 4 showed the highest accumulation in lysosomes, whereas a decrease in lysosome accumulation was found with the increase in log *P* for values higher than 4. Thus, the optimal combination and appropriate control of these two variables could be pivotal in the rational design of leishmanicidal agents, whose goal is to guarantee quinoline accumulation within the phagolysosome. Furthermore, the incorporation of the lipophilic group, which provides a general log *P* ∼ 1–4 to the molecule, seeks to facilitate the penetration of the quinoline drug through the lipophilic phagolysosome membrane, whereas the incorporation of a basic moiety, which provides *pK*_*a*_ ∼ 4–10 (*pK*_*a1*_ and *pK*_*a2*_ correspond to the quinolinic and alkyl amine chain) to the molecule, seeks to guarantee the accumulation inside the lysosome through an “ion trapping” mechanism that involves the capture of the molecule by generating a polar and cationic form through the protonation of the basic moieties in an acidic environment, as depicted in Scheme 1.

## Lipophilic and basic groups and the leishmanicidal response in quinolines

**FIGURE 1:**
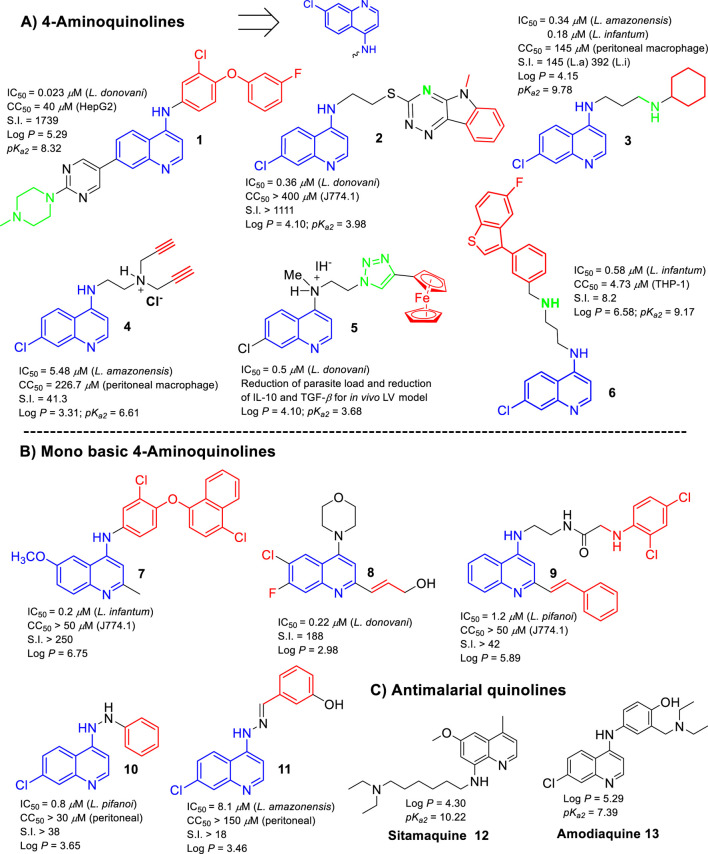
Structures of the most promising leishmanicidal quinoline compounds. **(A)** 4-Aminoquinolines; **(B)** monobasic 4-aminoquinolines; **(C)** antimalarial quinolines.

The present section seeks to correlate the role of lipophilicity and basicity with leishmanicidal activity based on the leishmanicidal response derived from an *in vitro* intracellular amastigote model. The analysis focused on the recent reviews published by Romero and Delgado (2025a); Del Carpio et al. (2025); Avanzo et al. (2025); Loiseau et al. (2022), which fully recompiles the leishmanicidal potential of 4-aminoquinolines, metal based-quinolines, antimalarial quinolines, and 2-substituted quinolines, respectively. The *pK*_*a*_ and log *P* values were estimated using ChemDraw software (ChemDraw Professional, 2016) and the SwissADME platform (Daina et al., 2017), respectively. Relevant cases are shown in Figure 1. Regarding the dibasic 4-aminoquinolines (Romero and Delgado, 2025a), it should be noted that most of the potent and selective 4-aminoquinolines (e.g., compounds **1**–**6**) are characterized by incorporating in their structures either a basic group (e.g., tertiary dialkylamine or *N*-heteroarene) or a lipophilic group (e.g., aryl or alkyl chain) that disclose appropriate or acceptable log *P* and *pK*_*a*_ parameters. For example, the most selective compound (compound **1**), which displayed an IC_50_ value of 0.023 µM against the amastigote of *Leishmania donovani* and a selectivity index (S.I.) of 1,739, showed an optimal *pK*_*a2*_ value of 8.32 and a discretely high log *P* value of 5.29. Compound **2**, which displayed a high anti-amastigote response against *L. donovani* (IC_50_ = 0.36 µM) and selectivity (S.I. > 1,111), showed an optimal log *P* of 4.10 and an appropriate *pK*_*a2*_ of 3.98. Meanwhile, compound **3**, which presented appropriate values of log *P* (4.15) and *pK*_*a2*_ (9.78), exhibited a high leishmanicidal response and selectivity against amastigote models of *Leishmania amazonensis* (IC_50_ = 0.34 µM; S.I. = 145) and *Leishmania infantum* (IC_50_ = 0.18 µM; S.I. = 392). Moreover, compound **4** showed moderate potency (IC_50_ = 5.48 µM) and selectivity (S.I. = 41) against the amastigote of *L. amazonensis*, which could be correlated more with its lower lipophilicity (log *P* = 3.31) than with its basicity because it presented an optimal *pK*_*a2*_ (6.61). Compound **5**, a quinoline-based metallic complex, was considered the most potent due to its excellent *in vitro* (IC_50_ = 0.5 vs. *L. donovani* amastigote) and *in vivo* responses (Del Carpio et al., 2025), with appropriate log *P* and *pK*_*a2*_ values of 4.10 and 3.68, respectively. Finally, of the analyzed 4-aminoquinolines (**1–6**), compound **6** (log *P* = 6.58; *pK*_*a2*_ = 9.17) was highly potent but highly toxic, which could be associated with its extremely high lipophilicity. In general, based on examples of 4-aminoquinolines, it has been consistently documented that the inclusion of either several basic or extended lipophilic moieties into the quinoline structure compromises the selectivity and leishmanicidal potency of the 4-aminoquinolines (Romero and Delgado, 2025a). Furthermore, it shows that the most active and selective leishmanicidal dibasic 4-aminoquinolines are characterized as having a log *P* ranging between 4 and 5.3 and a *pK*_*a2*_ ranging between 4 and 9.

Regarding the monobasic 4-aminoquinolines (with a *pK*_*a1*_ ∼4 by quinolinic nitrogen), some compounds (e.g., compounds **7**, **8**, **9**, **10**, and **11**) exhibited good anti-amastigote effects in a similar range to that of the most active 4-aminoquinolines mentioned above, but they were less selective. This indicates that the selectivity of these compounds appears to have a correlation with its lipophilic characteristic. For example, the most active monobasic quinoline (compound **7**), which showed an excellent potency against *L. infantum* (IC_50_ = 0.20 µM) and a good selectivity (S.I. ≥ 250), presented a high log *P* value of 6.75. The second most selective compound of this group (compound **8**) (S.I. = 188; IC_50_ = 0.22 µM vs. *L. donovani*) (Loiseau, et al., 2022) presented a log *P* value of 2.98. In this case, the inclusion of the halogens (F and Cl) and the morpholine moiety in the quinoline core could be essential to conveniently modulate the log *P* and *pK*_*a*_ magnitudes for favoring the accumulation inside the macrophage phagolysosome in the infected model. Another potent (IC_50_ = 1.20 µM vs. *Leishmania pifanoi*) and selective (S.I. > 42) compound (compound **9**) exhibited a high log *P* of 5.89. Meanwhile, compounds **10** (log *P* = 3.46) and **11** (log *P* = 3.65), which showed a log *P* < 4, exhibited more limited leishmanicidal activities and selectivities.

Finally, analyzing the leishmanicidal response of antimalarial quinolines (Avanzo et al., 2025), lipophilic amodiaquine (AQ), sitamaquine (SQ), mefloquine, and tafenoquine bear a second basic group and display the highest anti-amastigote response against *Leishmania* spp. However, the high cytotoxicity of mefloquine and tafenoquine reflects the importance of the selection of the type of lipophilic chain and its location on the quinoline ring. Furthermore, SQ (log *P* = 4.30; *pK*_*a2*_ = 10.22) and AQ (log *P* = 5.29; *pK*_*a2*_ = 7.39) were considered the most selective and potent antimalarial quinolines against the intracellular amastigote, which could be associated with their optimal *pK*_*a2*_ and log *P* values. Further reports have demonstrated that SQ is able to accumulate in membranous organelles such as acidocalcisomes (López-Martín et al., 2008) and parasite mitochondria (Vercesi and Docampo, 1992; Vercesi et al., 2000). This last finding supports that the internalization of the SQ into these organelles could be favored by their appropriate *pK*_*a2*_ and log *P* parameters.

## Conclusion

In summary, the present opinion article introduces the importance of the phagolysosome and its mechanism to favor its drug accumulation as pivotal concepts in the design of potent and selective leishmanicidal agents, which are applicable to the design of not only quinolines but also other types of leishmanicidal compounds. Based on the reported cases, it can be inferred that the high anti-amastigote response and selectivity of the quinoline compounds could be favored by their appropriate log *P* and *pK*_*a*_ parameters, which seeks to facilitate their transmembrane penetration and lysosome accumulation via “ion trapping.” It should be noted that the dibasic quinolines tend to generate more active and selective leishmanicidal compounds than monobasic quinolines and even more than tri- or polybasic quinolines. Within the dibasic quinolines, mainly those based on 4-aminoquinolines, the most potent and selective compounds are characterized as having *pK*_*a2*_ ranging between 6 and 9 and log *P* values ranging from 4 to 6. Meanwhile, the most promising monobasic quinolines presented, in general, log *P* values higher than 3 and lower than 6.5, with some exceptions, such as compound **8**, in which incorporation of halogen and morpholine moieties could be key to conveniently modulate the lipophilicity and basicity of the quinoline for good penetration and accumulation inside the phagolysosome.

In general, the ionization and lipophilic parameter requirements to achieve a good leishmanicidal response in the quinoline compounds are in good concordance with Trapp’s and Marceau’s findings, where a compound with a higher lipophilicity (log *P* ranging from 4 to 6) than expected for typical lysosome accumulation (log *P* ranging between 1 and 4) is still considered for lysosomal targeting. Probably, the combination of both factors under appropriate magnitudes could be highly required for the design of potent and selective leishmanicidal agents for macrophage phagolysosome targeting. However, there is an urgent need for further studies either to demonstrate its effective accumulation into the macrophage phagolysosome or to elucidate the most appropriate parameter magnitudes to achieve a good phagolysosome accumulation and control of the selectivity in the design of quinoline-based drugs and other types of leishmanicidal agents based on a screening study.
